# Factors associated with social casino gaming among adolescents across game types

**DOI:** 10.1186/s12889-018-6069-2

**Published:** 2018-10-11

**Authors:** Livia Veselka, Rochelle Wijesingha, Scott T Leatherdale, Nigel E Turner, Tara Elton-Marshall

**Affiliations:** 1 Centre for Addiction and Mental Health, Institute for Mental Health Policy Research, 100 Collip Circle, Suite 200, London, ON N6G 4X8 Canada; 2 Department of Sociology, McMaster University, Hamilton, ON Canada; 3 School of Public Health and Health Systems, University of Waterloo, Waterloo, ON Canada; 4 Dalla Lana School of Public Health, University of Toronto, Toronto, ON Canada; 5 Department of Epidemiology and Biostatistics, Western University, London, ON Canada

**Keywords:** Adolescent, Social casino games, Simulated gambling, Poker, Slots, Facebook

## Abstract

**Background:**

With the proliferation of social casino games (SCGs) online, which offer the opportunity to gamble without monetary gains and losses, comes a growing concern regarding the effects of these unregulated games on public health, particularly among adolescents. However, given the limited research pertaining to SCG use, little is currently known about the manner in which adolescents engage with this new gambling medium. The present study aims to identify the factors that characterize adolescent social casino gamers, and to determine whether these factors differ by SCG type. Moreover, the study examines the extent to which social casino gaming is associated with monetary gambling and problem gambling in this cohort.

**Method:**

Data were obtained from students in Grades 9 to 12 (*n* = 10,035) residing in the Canadian provinces of Ontario, Saskatchewan, and Newfoundland and Labrador. Participants completed the Youth Gambling Survey (YGS), which is a supplementary instrument administered alongside the Canadian Student Tobacco, Alcohol and Drugs Survey (CSTADS). Logistic regression was used to assess the factors associated with SCG play.

**Results:**

Overall, 12.4% of respondents reported having participated in SCGs in the past three months. Compared to adolescents who did not report playing SCGs, SCG players were typically more likely to participate in monetary gambling activities, and were more prevalently classified as problem gamblers of low-to-moderate severity or high severity. Although profiles of SCG players differed across SCG game types, factors significantly associated with the playing of SCGs were gender, weekly spending money, having friends and parents who gamble, and screen time. It was also shown that current smokers were significantly more likely to participate in simulated slots online relative to adolescents who did not play SCGs.

**Conclusion:**

Significant associations exist between SCG play, monetary gambling, and problem gambling among adolescents. Gambling intervention efforts directed at this population should aim to identify personal and environmental factors associated with social casino gaming, and should be tailored to different types of SCGs.

## Background

Advancements in technology have fueled the expansion of gambling activities beyond land-based venues and into widely accessible online formats. This growth has resulted in the development of social casino games (SCGs)—online games featuring a gambling theme that are available through standalone websites, social networking platforms, and mobile-device applications [1, 2]. SCGs closely resemble gambling activities in that they require players to stake bets on outcomes governed by elements of chance in an effort to win rewards [3, 4]. These games include, but are not limited to casino table games, slots, poker, lotto, bingo, and sports betting. SCGs differ fundamentally from monetary gambling, however. Specifically, while bets and winnings in SCGs take the form of virtual currency, which holds no value outside of the games, and which cannot be converted into money, monetary gambling is defined by bets and winnings that take the form of currency or other items of monetary value [2]. Further, given that SCGs are commonly built on a “freemium” revenue model, whereby the full version of a game is provided to players at no cost, players also do not pay to access SCGs [5]. Players can, however, make small-sum monetary transaction within SCGs, referred to as “microtransactions”, which allow them to extend play when they exhaust initial seed credits, to speed up play, or to purchase cosmetic or functional virtual goods that enhance their gaming experience [1, 6]. These microtransactions represent the main revenue source for SCGs. Given the absence of monetary betting and rewards in SCGs, these games are not legally classified as gambling activities, and are therefore unregulated [7].

Social casino gaming represents a popular form of entertainment. Globally, about 170 million individuals play SCGs monthly [8], and approximately 81 million individuals play SCGs on a daily basis [9]. While preliminary evidence suggests that the average age of SCG players ranges from approximately 30 to 45 years [2, 6, 10], these estimates are based on investigations of adult samples only, and therefore poorly represent the full breadth of players who engage in these games. Studies of adolescent use of SCGs reveal that between 10 to 32% of adolescents have participated in social casino gaming at some point in their lives [2, 11–15]. These estimates are in line with prevalence rates reported for SCG play among adults [10, 11]. Therefore, adolescents represent a cohort with substantial exposure to and interest in SCGs.

Despite the availability of best practice principles set forth by the International Social Games Association [16], social casino gaming is largely unregulated. As a result, SCGs typically do not enforce recommended age restrictions that restrict SCG access to individuals younger than 18 years of age, thereby providing ready access to adolescents who choose to take part in these games [17]. This engagement in simulated gambling games by young individuals is deemed potentially problematic because it may encourage an early transition into monetary gambling [2, 14, 18, 19]. Specifically, by normalizing gambling behaviours, offering a training ground through which gambling-related skills and habits are developed, and instilling an excitement for gambling activities that can only be augmented through higher-stakes waging and winning, SCGs may act as a gateway to gambling for adolescents [2, 17, 19–22]. Additionally, studies show that early exposure to gambling activities, including participation in SCGs at a young age, is a risk factor for the development of future problem gambling [23, 24], whereby individuals experience considerable negative consequences and personal distress as a result of their gambling activities [25]. Therefore, the availability of SCGs to adolescents may facilitate problem gambling tendencies among these individuals, and may ultimately have implications for their mental health and well-being. As a result, social casino gaming represents a potential public health issue.

Existing studies have reported that adolescents who play SCGs are more likely to engage in monetary gambling, and are more likely to endorse indicators of pathological gambling in comparison to SCG non-players [14, 26, 27]. Most notably, longitudinal studies of adolescents residing in Northern Germany [28] and the province of Quebec, Canada [26] have reported that participation in simulated versions of gambling games is a significant predictor of subsequent monetary gambling. Although a mirroring of these links has also been noted between among adults [20, 21, 29], it should be noted that adolescents may be particularly susceptible to the negative impacts of SCG use. In support, Gainsbury, King et al. (2015) observed that 28% of adolescents versus 17% of adults in their study had increased their participation in monetary gambling as a direct result of SCG use. Additionally, 33% of adolescents and only 15% of adults in the study endorsed the erroneous belief that SCG play will lead to increased success in subsequent gambling activities. As a result, adolescents appear to be more likely than adults to carry inaccurate perceptions of SCGs, and to use SCGs as a springboard for further gambling activities. Consequently, they are a vulnerable population in the study of SCG use that warrants more comprehensive investigation.

### Characteristics of social casino gamers

Despite the fact that adolescents are avid users of SCGs, and appear to be particularly susceptible to the negative consequences of these games, little is known about the characteristics of adolescent social casino gamers. In fact, studies of the environmental and personal factors associated with SCG play have focused almost exclusively on adult samples [10, 30, 31], and therefore potentially relevant variables specific to younger cohorts have largely been omitted from SCG investigations. Establishing the features that are typical of adolescent social casino gamers is critical to informing targeted intervention strategies, and to identifying at-risk sub-groups within the adolescent population.

In studies of adult samples, researchers have identified a number of factors that are more characteristic of SCG players versus non-players [10, 30]. Specifically, researchers have noted that male SCG players are more likely to engage in competitive SCGs, particularly poker, whereas female SCG players show a preference for online gaming-machine games (e.g., slots), and for gambling games that promote social interactions [10]. These results are in line with studies of online and land-based monetary gambling reported among adults and adolescents [32–34]. Additionally, adult SCG players are more likely to smoke tobacco on a daily basis, and are more likely to have used illicit drugs in the past 12 months in comparison to individuals who do not use SCGs [10]. Lastly, compared to adults who do not take part in social casino gaming, adults who play SCGs are more likely to be employed full-time, but are also more likely to be unemployed or reliant on a disability pension [10]. Empirical research is needed to determine whether these same characteristics that defines adult SCG users are also applicable to adolescents who take part in social casino gaming.

Although a number of the factors that are characteristic of adult SCG players are also relevant to adolescents, it is plausible that additional characteristics beyond those identified for adults are related to SCG play among adolescents. These additional correlating factors with SCG play are possible, given that adolescents are distinct from adults developmentally, and they exhibit gambling tendencies and preferences that are unique from those of adults [35, 36]. Studies of monetary online gambling among adolescents may provide preliminary insight into the manner in which these potentially overlooked variables are related to social casino gaming, given that SCGs and monetary online gambling games are typically used by individuals with similar sociodemographic profiles [10]. A key factor that may have an impact on SCG use among adolescents is peer and parental transmission of behaviours and attitudes pertinent to gambling [37, 38]. Studies carried out in Canada, the United Kingdom, and Hong Kong have shown that adolescents who have close friends or parents who engage in monetary gambling are more likely to participate in monetary online gambling [15, 39–41]. This role of close others in explicitly or tacitly encouraging gambling activities may be especially pertinent to social casino gaming, which often involves the sharing of SCG scores or the promotion of SCG play with one’s online social networks through social media websites [1].

Academic performance is another factor not studied among adult SCG users that may be relevant to an understanding of social casino gaming among adolescents, particularly due to the centrality of school activities to adolescent lives [42]. Existing studies of online gambling show that poor academic performance, as reflected by lower grades in school, is predictive of monetary online gambling among adolescents [15, 43, 44]. Further, academic problems, such as missed classes, poor study habits, and failure to submit work, are also significantly associated with more pervasive monetary online gambling [41]. These same patterns of effects may further extend to SCG use.

Sedentary behaviour, exhibited by a lack of physical activity, may also be a factor relevant to adolescent SCG players. Although sedentary behaviour has been largely overlooked in studies of monetary gambling behaviours in general, some existing evidence suggests that more substantial periods of inactivity as well as indicators of poor physical health, such as obesity, are positively associated with a propensity toward monetary gambling across all age groups [45, 46]. Additionally, it has been shown that sedentary behaviour is particularly prominent during adolescence, with Canadian adolescents typically spending over 8 h daily engaging in sedentary activities, primarily those involving screens [47–49]. Although a pervasiveness in screen time is harmful on its own [50], it may also increase exposure to online gaming and gambling, and may subsequently result in greater SCG play among this cohort.

An additional factor that may be associated with social casino gaming among adolescents is binge-drinking—the tendency to engage in the heavy consumption of alcohol over a short period of time with the intention of becoming intoxicated [51]. Binge-drinking has been show to increase during adolescence [52], and it has been linked to risky activities, including illicit drug use, tobacco use, and physical aggression [53, 54]. Previous studies of adolescents have further reported a significant association between binge-drinking and the risky activity of monetary gambling. Specifically, it has been shown that adolescents with a history of gambling are more likely to have experienced episodes of binge-drinking in the past year [55]. Binge-drinking is also significantly associated with at-risk and pathological gambling among adolescents [56, 57]. Theories of deviance suggest that a general propensity toward risk-taking may explain the typical co-occurrence between alcohol misuses and other risky activities, including gambling, whereby adolescents who seek stimulation and short-term and immediate gratification tend to engage in multiple problem behaviours [58, 59]. If this behavioural pattern extends to social casino gaming, then it is feasible that binge-drinking may also be associated with SCG play among adolescents.

### Present study

The present study represents one of the first empirical analyses of the characteristics defining adolescent social casino gamers. It is also one of the first studies to examine the factors associated with social casino gaming across different types of SCGs: poker, slots, and SCGs hosted on the social media site Facebook. Poker and slots represent the most popular SCGs among adult samples [10, 17], and Facebook is the most popular social media site for SCG play [21]. The differentiation between SCG types in the present analysis was deemed important given that previous research on monetary gambling has demonstrated that individuals who engage in different types of gambling activities are typically defined by unique characteristics and tendencies [40, 60]. These same effects may also be applicable to SCGs. In addition to exploring these new effects, the present study aims to replicate previously reported associations between SCG use, monetary gambling, and problem gambling among adolescents. In pairing these replication efforts with assessments of SCG player characteristics, the aim is to contribute to a better understanding of the manner in which SCG play is related to monetary gambling among adolescents, and to build upon existing longitudinal studies on this topic. Use of SCGs in the past three months is assessed in the present study to capture current participation rather than lifetime participation in social casino gaming. Further, a large and representative sample of adolescents from three Canadian provinces is examined in the present study, thereby overcoming a limitation of the majority of Canadian studies of adolescent gambling that have primarily relied upon convenience samples recruited from major cities [61].

Based on existing literature, it is predicted that, among adolescents, males will be more likely to play the SCG of poker, whereas females will be more likely to play the SCG of slots [10]. Further, it is hypothesized that those who have played SCGs in the past three months will be more likely to report: being a current smoker, having access to either very high or very low disposable income, having parents or close peers who gamble, achieving lower grades in school, leading a more sedentary lifestyle, binge-drinking, and engaging in monetary gambling activities [10, 15, 44]. Additionally, based on existing findings, it is expected that higher-severity problem gambling will be observed among adolescents who have played SCGs in the past three months versus those who have not [14, 26]. Specific hypotheses regarding the manner in which factors associated with SCG play may differ across game types have not been put forth due to the paucity of research on this subject.

## Method

### Design

Participants in the study were 10,035 secondary-school students in Grades 9 to 12, ranging in age from 13 to 19 years. Participants completed the Youth Gambling Survey (YGS; [62]), a supplementary instrument administered alongside the Canadian Student Tobacco, Alcohol and Drugs Survey (CSTADS), formerly the Youth Smoking Survey (YSS) [63, 64]. Students were recruited to complete the YGS through stratified multistage sampling that yielded provincially representative samples from three Canadian provinces: Ontario, Saskatchewan, and Newfoundland and Labrador. All school boards, schools, and students who took part in the YSS in these provinces were also eligible to complete the YGS. Overall, a total of 15,269 students across the three provinces were eligible to take part in the YGS, and the response rate was 66%. The YGS was administered in both English and French following the YSS questionnaire, and it was completed by participating students in their classrooms during school hours. The administration of both the YSS and the YGS took approximately 20–30 min within each class. Data collection occurred in the years 2012–2013, and therefore it preceded the legalization of online gambling that took place in Ontario in January, 2015. Additional details regarding the YSS can be found at: https://uwaterloo.ca/canadian-student-tobacco-alcohol-drugs-survey/

Ethics approval for both the YSS and the YGS was granted by the University of Waterloo Research Ethics Office. Parental consent was obtained for all participating students via either an active permission protocol or an active information/passive permission protocol, based on the requirements of the corresponding school board or school. On the day of participation, all students were notified of their right to decline participation in the study.

### Measures

#### Social casino gaming

Three items were used to measure whether participants have played SCGs, consistent with previous studies [15, 65]. Specifically, participants were asked how often they played the following games for fun (no money): Internet poker, Internet slots, gambling games on Facebook. Response options consisted of: “not in the past 3 months”, “about once per month”, “2-3 times per month”, “about once per week”, “2-6 times per week”, “daily”. Participants were categorized as being SCG players for each game type if they indicated that they played a given game with any frequency in the past three months. In cases where participants reported having played multiple types of SCGs in the past three months, their responses were assessed under each game-type category that they endorsed. Consequently, it was possible for a single participant to be represented under each of the three game-type categories in the study.

#### Modality of monetary gambling

Land-based gamblers were participants who reported gambling for money or for something of value at least once in the past three months through one or more of the following gambling activities: (1) lottery tickets; (2) instant-win or scratch tickets; (3) cards; (4) board games or dice; (5) video lottery terminals; (6) slot machines not online; (7) arcade or video games; (8) sports select; (9) sports pools or games not online; (10) horse races; (11) performance in games of skill (e.g., pool, golf, bowling, darts) or other activities; (12) a dare or challenge; (13) bingo.

Online gamblers were participants who indicated that they had gambled for money or for something of value at least once in the past three months on any of the following three activities: (1) Internet poker; (2) sports pools or games online; (3) slot machines online. In the current sample, only 1.9% of respondents (*n* = 74) gambled solely online. Therefore, our sample of online gamblers (*n* = 833) includes those individuals who gambled exclusively online as well as those who gambled both online and in land-based gambling.

Respondents were coded as non-gamblers if they indicated that they did not gamble in the past three months on any of the activities listed above.

#### Problem gambling severity

The severity of problem gambling experienced by participants was measured using the Gambling Problem Severity Subscale (GPSS) of the Canadian Adolescent Gambling Inventory (CAGI) [66]. The CAGI is the seminal measure developed to assess the behavioural component of gambling among adolescents, and it has demonstrated sound psychometric properties in previous studies using adolescent samples [67–69]. The GPSS consists of nine items assessing behaviours pertinent to problem gambling that occurred in the past three months, which have psychological, social, financial, and inhibitory consequences. Participants respond to each item by indicating the frequency with which they engage in the target behaviour using a Likert-type scale on which higher scores indicate greater frequency. To calculate gambling severity, ratings on the GPSS are summed, and the total number of items that were answered is subtracted from the sum. Gambling severity scores are then categorized as follows: GPSS ≤1 = no problem gambling, GPSS 2 to 5 = low to moderate gambling severity, GPSS 6 to 27 = high gambling severity.

#### Parent or friend who gambles

Respondents were asked to indicate if any of their parents, step-parents, or guardians gambled for money. Responses were coded as “yes”, “no” and “don’t know or not stated”.

Participants also indicated how many of their closest friends gambled for money. Responses were then coded as “yes”, “no” and “don’t know or not stated” to indicate whether or not they had one or more close friends who gambled.

#### Substance use

Respondents were classified as current smokers if they reported smoking at least 100 cigarettes in their lifetime, and if they indicated that they had smoked in the past 30 days. Respondents were classified as former smokers if they reported smoking at least 100 cigarettes in their lifetime, and if they indicated that they had not smoked in the past 30 days. Respondents were classified as non-smokers if they reported having never smoked or if they indicated that they had smoked fewer than 100 cigarettes in their lifetime. For the purposes of analysis, smoking status was represented as a dichotomous variable characterized by two levels: current smokers and former/non-smokers. The 100-cigarette criterion for determining smoking status is an established criterion in tobacco research [70, 71], and it has been used reliably in previous studies of tobacco use [72, 73].

To assess binge-drinking, participants responded to an item that asked whether they had consumed five drinks of alcohol or more on one occasion in the past 12 months. This definition of binge-drinking is consistent with previous studies of adolescent alcohol consumption [74, 75]. Responses were coded as “never or not in the last year”, “yes, in the last year”, and “don’t know or not stated”.

#### School performance

To measure school performance, respondents were asked to select the marks they typically receive in school. Responses were then coded as “mostly As”, “mostly As and Bs”, “mostly Bs and Cs”, “mostly Cs or lower”, and “not stated”.

#### Screen time

Sedentary tendencies were measured through an assessment of daily screen time. Specifically, respondents were asked to report how many hours per day, on average, they spend doing the following: (1) watching/streaming TV shows or movies; (2) playing video or computer games; (3) surfing the Internet. The estimates provided by each participant across the measured activities were summed to create a single continuous variable of screen time. Due to the positive skew of the summed scores, a natural logarithmic transformation was employed to make the measure more symmetrical.

#### Sociodemographic variables

Participants were asked to report on their gender (male/female), province of residence (Newfoundland and Labrador, Ontario, Saskatchewan), grade (9–12), and weekly spending money. Weekly spending money was measured by asking participants how much money they receive or earn each week to spend on themselves or to save. Responses were categorized as: “$0 to $20”, “$21 to $100”, “more than $100”, “don’t know/missing”.

### Data analysis

Logistic regression analyses were employed to examine the factors associated with each type of social casino gaming: poker, slots, and SCGs on Facebook. Bootstrap survey weights were used in order to account for the sampling design. The statistical package STATA 12.0 was used for all analyses.

## Results

Sample characteristics for all adolescents in the present study (*n* = 10,035) are reported in Table 1. Overall, 9.1% of adolescents in the three Canadian provinces assessed indicated that they had played the SCG of poker in the past 3 months, 5.0% reported participating in the SCG of slots in the past 3 months, and 9.0% stated that they had played SCGs on Facebook in the past three months. Further, 32.3% of adolescents indicated that they had taken part in land-based gambling in the past three months, and an additional 9.3% indicated that they had gambled online for money in the past three months. Although the majority of the sample did not report symptoms of problem gambling, 4.2% of adolescents in the three provinces surveyed endorsed items indicative of low to moderate problem gambling severity, and 2.2% reported behaviours reflective of high problem gambling severity.

**Table 1:** Sample Characteristics (*n* = 10,035), Youth Gambling Survey (YGS; Canada, 2012–2013)

	Unweight (*n*)	Weighted (%)
SCG poker (past 3 months)		
Yes	831	9.1
No	8415	90.9
SCG slots (past 3 months)		
Yes	498	5.0
No	8711	95.1
Facebook SCGs (past 3 months)		
Yes	796	9.0
No	8440	91.0
Monetary gambling in past 3 months		
Did not gamble	5902	58.4
Land-based gambling	3095	32.3
Online gambling	833	9.3
Problem gambling severity		
No problem gambling	8861	93.5
Low to moderate	338	4.2
High	216	2.2
Gender		
Male	4937	49.3
Female	5098	50.7
Grade		
9	2635	22.6
10	2714	23.5
11	2403	23.1
12	2283	30.8
Province		
Ontario	3892	89.4
Newfoundland & Labrador	2588	3.0
Saskatchewan	3555	7.5
Weekly spending money		
$0–$20	4018	41.9
$21–$100	2537	22.9
> $100	1610	16.0
Don’t know/not stated	1870	19.2
Smoking status		
Former smoker/never smoked	9187	92.6
Current smoker	848	7.4
Binge drinking		
Never/not in last year	5815	59.2
Yes, in the last year	3997	38.6
Don’t know/not stated	223	2.3
Friend(s) who gamble		
No	7560	73.1
Yes	1929	21.4
Not stated	546	5.5
Parent(s) who gambles		
No	5260	51.8
Yes	2523	25.0
Don’t know/not stated	2252	23.2
School performance		
Mostly As	3313	29.9
Mostly As and Bs	4296	47.5
Mostly Bs and Cs	1632	16.0
Mostly Cs or lower	613	4.3
Not stated	181	2.4
Screen time	9597	*Mean* = 3.6 hours *Median* = 3.0 hours

*Note*: *SCG* = social casino game. It is evident that the screen time variable is skewed, and therefore a natural logarithmic transformation was employed to make the measure more symmetrical in subsequent analyses

Among the 12.4% of respondents who reported having participated in any SCG in the past three months (*n* = 1204), 57.3% of SCG players had played only one type of SCG, while nearly 1 in 5 SCG players (19.4%) had played all three game types. Poker was the most popular SCG, with 67.6% of all SCG players indicating that they had played this SCG in the past three months. Poker was also the most popular game among SCG players who reported playing only one type of SCG in the timeframe assessed, with 29.1% of SCG players stating that they had played only this game in the past three months. Additionally, 23.4% of SCG players indicated that they had played only SCGs on Facebook in the past three months, and 4.8% stated that they had played only the SCG of slots in the past three months. Further, 23.4% of SCG players indicated that they had played two SCGs in the past three months. Specifically, 12.7% of SCG players indicated that they had played the SCG of poker and Facebook SCGs, 6.4% reported that they had played the SCG of poker and the SCG of slots, and 4.3% stated that they had played Facebook SCGs and the SCG of slots in the preceding three months.

### Problem gambling among SCG players versus non-players across game types

**Fig. 1:**
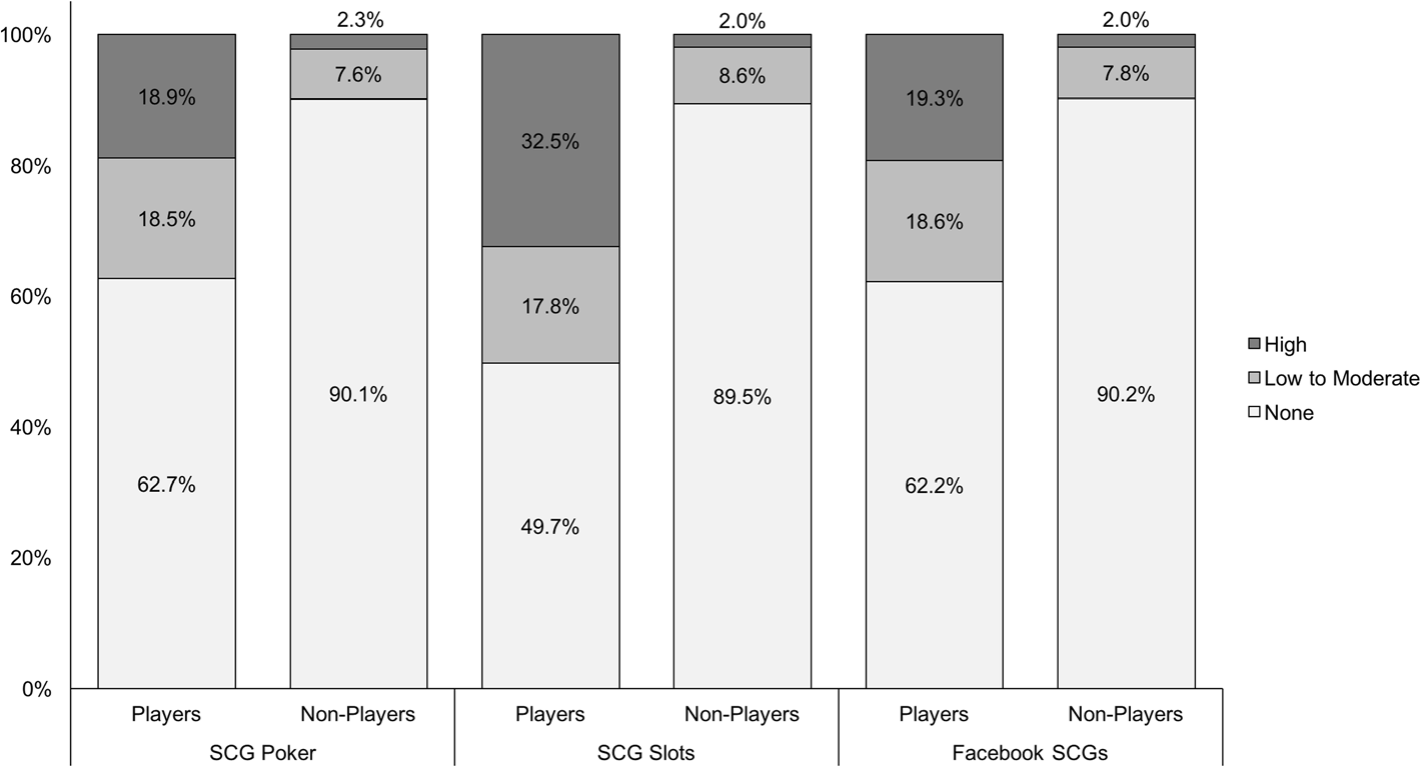
Severity of Problem Gambling by Social Casino Game Type Among Current Adolescent Gamblers (*n* = 3928)

Problem gambling prevalence among current gamblers was compared between SCG players and SCG non-players across different types of SCGs (see Fig. 1). Across all game types assessed, a significantly larger proportion of SCG players versus non-players were classified as exhibiting gambling problems of low-to-moderate severity or high severity. In contrast, the vast majority of SCG non-players across all games types did not endorse items indicative of problem gambling. Specifically, for the SCG of poker, 18.5% of players versus 7.6% of non-players were categorized as having gambling problems of low-to-moderate severity, and 18.9% of players versus 2.3% of non-players were categorized as having gambling problems of high severity (*χ*^2^ = 64.07, *p* < 0.001). For the SCG of slots, 17.8% of players versus 8.6% of non-players were classified as having gambling problems of low-to-moderate severity, and 32.5% of players versus 2.0% of non-players were classified as having gambling problems of high severity (*χ*^2^ = 186.00, *p* < .001). Lastly, for SCGs accessible via Facebook, 18.6% of players versus 7.8% of non-players were shown to have gambling problems of low-to-moderate severity, and 19.3% of players versus 2.0% of non-players were shown to have gambling problems of high severity (*χ*^2^ = 111.59, *p* < 0.001). Overall, adolescents who reported playing the SCG of slots exhibited the highest proportion of high-severity gamblers.

### Factors associated with social casino gaming

Logistic regression analyses were conducted to assess the factors associated with participation in three different types of social casino gaming in the past three months (Table 2). Separate models were examined for adolescents who reported playing SCG poker (Model 1), SCG slots (Model 2), and SCGs games on Facebook in the past (Model 3), compared to adolescents who did not report playing any type of SCGs in the past three months.

**Table 2:** Logistic Regression: Social Casino Game (SCG) Players versus Non-Players Across Game Type (*n* = 10,035)

	Model 1: SCG poker Adjusted Odds Ratio, 95% CI	Model 2: SCG slots Adjusted Odds Ratio, 95% CI	Model 3: Facebook SCGs Adjusted Odds Ratio, 95% CI
Gender			
Male	Reference	Reference	Reference
Female	**0.34 (0.26–0.45),** ***p*** **< 0.001**	0.89 (0.68–1.16), *p* = 0.387	0.83 (0.64–1.06), *p* = 0.134
Grade			
9	Reference	Reference	Reference
10	0.99 (0.73–1.33), *p* = 0.932	0.96 (0.56–1.66), *p* = 0.888	1.35 (0.85–2.12), *p* = 0.199
11	0.87 (0.58–1.30), *p* = 0.502	0.82 (0.47–1.43), *p* = 0.487	0.88 (0.56–1.36), *p* = 0.553
12	1.04 (0.70–1.54), *p* = 0.853	0.62 (0.35–1.09), *p* = 0.097	0.66 (0.44–1.01), *p* = 0.054
Province			
Ontario	Reference	Reference	Reference
Newfoundland & Labrador	0.98 (0.80–1.19), *p* = 0.811	1.18 (0.87–1.61), *p* = 0.281	0.85 (0.59–1.22), *p* = 0.378
Saskatchewan	0.90 (0.68–1.19), *p* = 0.463	0.84 (0.56–1.26), *p* = 0.393	0.70 (0.48–1.04), *p* = 0.076
Weekly spending money			
$0–$20	Reference	Reference	Reference
$21–$100	1.27 (0.84–1.92), *p* = 0.254	1.32 (0.79–2.20), *p* = 0.289	**1.62 (1.09–2.41),** ***p*** **= 0.018**
> $100	**1.70 (1.22–2.36),** ***p*** **= 0.002**	**2.83 (1.81–4.42),** ***p*** **< 0.001**	**1.71 (1.05–2.79),** ***p*** **= 0.032**
Don’t know/not stated	1.18 (0.84–1.67), *p* = 0.328	**2.13 (1.40–3.24),** ***p*** **< 0.001**	1.54 (0.94–2.52), *p* = 0.084
Smoking status			
Former smoker/never smoked	Reference	Reference	Reference
Current smoker	1.36 (0.72–2.55), *p* = 0.341	**2.11 (1.17–3.80),** ***p*** **= 0.013**	1.42 (0.92–2.18), *p* = 0.114
Binge drinking			
Never/not in last year	Reference	Reference	Reference
Yes, in the last year	0.94 (0.67–1.31), *p* = 0.716	0.75 (0.52–1.08), *p* = 0.126	0.87 (0.62–1.21) *p* = 0.397
Don’t know/not stated	0.66 (0.32–1.37), *p* = 0.266	0.72 (0.24–2.12), *p* = 0.551	0.41 (0.16–1.07), *p* = 0.068
Friend(s) who gamble			
No	Reference	Reference	Reference
Yes	1.35 (0.94–1.93), *p* = 0.101	**1.93 (1.45–2.56),** ***p*** **< 0.001**	**1.89 (1.44–2.48),** ***p*** **< 0.001**
Not stated	**3.86 (1.72–8.65),** ***p*** **= 0.001**	**3.76 (1.37–10.27),** ***p*** **= 0.010**	**2.39 (1.02–5.59),** ***p*** **= 0.044**
Parent(s) who gambles			
No	Reference	Reference	Reference
Yes	**1.55 (1.16–2.09),** ***p*** **= 0.004**	**1.69 (1.17–2.44),** ***p*** **= 0.005**	**1.51 (1.13–2.00),** ***p*** **= 0.005**
Don’t know/not stated	0.94 (0.74–1.21), *p* = 0.640	1.01 (0.68–1.50), *p* = 0.965	**1.49 (1.15–1.93),** ***p*** **= 0.002**
School performance			
Mostly As	Reference	Reference	Reference
Mostly As and Bs	1.21 (0.79–1.87), *p* = 0.381	1.15 (0.73–1.81), *p* = 0.556	1.18 (0.93–1.49), *p* = 0.169
Mostly Bs and Cs	1.50 (0.91–2.50), *p* = 0.115	1.44 (0.78–2.68), *p* = 0.247	1.42 (0.94–2.16), *p* = 0.099
Mostly Cs or lower	1.60 (0.99–2.58), *p* = 0.058	1.60 (0.83–3.08), *p* = 0.157	1.40 (0.88–2.22), *p* = 0.150
Not stated	1.40 (0.55–3.60), *p* = 0.480	2.05 (0.80–5.26), *p* = 0.136	1.86 (0.79–4.40), *p* = 0.158
Screen time (log)	1.09 (0.83–1.43), *p* = 0.540	**2.18 (1.61–2.95),** ***p*** **< 0.001**	**1.54 (1.24–1.91),** ***p*** **< 0.001**
Monetary gambling in past 3 months			
Did not gamble	Reference	Reference	Reference
Land-based gambling	1.27 (0.85–1.91), *p* = 0.240	**1.50 (1.03–2.19),** ***p*** **= 0.035**	**1.64 (1.25–2.15),** ***p*** **< 0.001**
Online gambling	**5.85 (3.66–9.35),** ***p*** **< 0.001**	**7.33 (4.73–11.36),** ***p*** **< 0.001**	**5.76 (3.88–8.54),** ***p*** **< 0.001**

*Note*: *SCG* = social casino game. Statistically significant findings appear in bold font

#### Model 1: SCG poker players versus SCG non-players

Females (OR = 0.34, 95% CI 0.26–0.45, *p* < 0.001) had significantly lower odds of playing the SCG of poker relative to males. Additionally, respondents who earned more than $100 per week (OR = 1.70, 95% CI 1.22–2.36, *p* < 0.01) were significantly more likely to play the SCG of poker than respondents who earned between $0 to $20 weekly. Lastly, adolescents who reported having a parent who gambles (OR = 1.55, 95% CI 1.16–2.09, *p* < 0.01), and those who had gambled online in the past three months (OR = 5.85, 95% CI 3.66–9.35, *p* < 0.001) were more likely to have played the SCG of poker.

#### Model 2: SCG slots players versus SCG non-players

Findings revealed that adolescents who earned more than $100 weekly (OR = 2.83, 95% CI 1.81–4.42, *p* < 0.001) had significantly greater odds of playing the SCG of slots than those who earned $0 to $20. Further, a significantly greater likelihood of playing the SCG of slots was observed among respondents who reported being current smokers (OR = 2.11, 95% CI 1.17–3.80, *p* < 0.05), among those who indicated that they had a close friend (OR = 1.93, 95% CI 1.45–2.56, *p* < 0.01) or parent who gambles (OR = 1.69, 95% CI 1.17–2.44, *p* < 0.01), and among those who reported more screen time on a daily basis (OR = 2.18, 95% CI 1.61–2.95, *p* < 0.001). In addition, adolescents who participated in land-based gambling (OR = 1.50, 95% CI 1.03–2.19, *p* < 0.05) or online gambling (OR = 7.33, 95% CI 4.73–11.36, *p* < 0.001) in the past three months were significantly more likely to indicate that they have played the SCG of slots, compared to individuals who did not gamble in the past three months.

#### Model 3: Facebook SCG players versus SCG non-players

Results indicated that, compared to individuals who earned $0 to $20 per week, those who earned $21 to $100 weekly (OR = 1.62, 95% CI 1.09–2.41, *p* < 0.05) and over $100 weekly (OR = 1.71, 95% CI 1.05–2.79, *p* < 0.05) had significantly greater odds of playing SCGs on Facebook. A greater likelihood of playing SCG games on Facebook was also noted for adolescents who indicated that they have a close friend (OR = 1.89, 95% CI 1.44–2.48, *p* < 0.001) or parent (OR = 1.51, 95% CI 1.13–2.00, *p* < 0.01) who gambles, and for adolescents who reported engaging in more daily screen time (OR = 1.54, 95% CI 1.24–1.91, *p* < 0.001). Lastly, results indicated that, compared to adolescents who had not gambled in the past three months, those who took part in land-based gambling (OR = 1.64, 95% CI 1.25–2.15, *p* < 0.001) and in online gambling (OR = 5.76, 95% CI 3.88–8.54, *p* < 0.001) during this same time period were significantly more likely to report also playing SCGs on Facebook.

## Discussion

The goal of the present investigation was to obtain a more comprehensive understanding of the factors associated with social casino gaming among adolescents across three types of SCGs. Results from the study revealed significant associations between social casino gaming, monetary gambling in online and land-based forms, and indicators of problem gambling. Additionally, findings identified key factors that distinguish adolescent social casino gamers from adolescents who do not play SCGs.

Overall, the proportion of adolescents who reported playing SCGs in the present study (12.4%) is in line with previous estimates of social casino gaming prevalence among both adolescent and adult samples [10]. It should be noted, however, that the proportion of adolescent SCG players observed in the present study may be an underestimate of social casino gaming prevalence, given that SCG play in the current study is restricted to the past three months. Further, consistent with previous studies of social casino gaming among adults the present findings showed that the most popular SCG among adolescents is simulated poker [10, 17].

### Social casino gaming and monetary gambling among adolescents

The present study revealed that, compared to individuals who did not play SCGs, adolescent SCG users were more likely to participate in both online monetary gambling and land-based monetary gambling across nearly all SCGs assessed. Previous investigations of SCG use among adolescents and adults have also reported similar findings [10, 20, 29]. Given the cross-sectional nature of the present study, it is not possible to know whether SCG play facilitates the transition to monetary gambling, whether adolescents who gamble seek out SCGs as a substitute for monetary gambling activities, or whether individuals who are already monetary gamblers choose to play SCGs because of the considerable similarities between the two activities [6, 11, 14, 20, 76]. However, recent longitudinal analyses conducted using adult and adolescent samples suggest that social casino gaming may indeed facilitate the crossover into monetary gambling [21, 26]. Consequently, it is plausible that social casino gaming may act as a prime for subsequent monetary gambling by facilitating the development of gambling habits that can be extended into monetary venues [17, 22]. This transition from SCG play into monetary gambling may be particularly relevant to some Canadian jurisdictions (i.e., Ontario) where monetary online gambling has been legalized. The legalization of monetary online gambling creates an environment in which gambling via the Internet is legitimized and accessible [77]. It further leads to the proliferation of advertising for online monetary gambling games, particularly within SCGs [23]. Consequently, adolescent SCG players residing in jurisdictions where online monetary gambling is legal may be particularly at-risk of pursuing this type of gambling given their familiarity with Internet-based gambling activities, and given their exposure to online-gambling advertisements. Further research is needed to examine the link between social casino gaming and online monetary gambling pre- and post-legalization of online gambling in relevant jurisdictions.

Interestingly, while players of SCG poker were significantly more likely than SCG non-players to take part in online monetary gambling, they did not differ significantly from SCG non-players in their tendency to seek out land-based monetary gambling. A potential explanation for this observation may be rooted in existing studies of typical gambling habits. Specifically, research has shown that individuals who gamble across different modalities tend to be consistent in the gambling activities they pursue [24, 26]. Among poker players, who typically identify strongly with the game of poker [26, 78], the tendency to remain dedicated to the game across various platforms may be particularly evident. In the case of adolescents who play the SCG of poker, however, a transition from social casino gaming communities into strictly monitored land-based monetary gambling venues may be difficult due to legal restrictions or the potentially intimidating nature of the poker-game environment. Instead, adolescent poker players interested in monetary gambling may more avidly seek out online monetary gambling opportunities that offer a less regulated and largely anonymous gambling environment [79, 80]. Further, the draw to online monetary poker among SCG poker players may be magnified by the popularity of the game on the Internet, and by the pervasive advertising for online poker that is typically embedded in SCGs [81]. These suggested effects represent an opportunity for further study, particularly in the context of SCGs.

### Social casino gaming and problem gambling among adolescents

Findings from the study revealed that, across all types of SCGs, a larger proportion of SCG players than non-players were classified as exhibiting signs of problem gambling. In contrast, adolescents who did not report playing SCGs in the past three months were predominantly classified as not being problem gamblers. These results are supportive of existing studies of adults and adolescents, in which social casino gaming has exhibited associations with problem gambling tendencies [2, 14, 27]. While the present results stem from a cross-sectional design, which precludes the identification of causal effects, it may be the case that individuals, and particularly adolescents, increase their likelihood of experiencing symptoms pertinent to problem gambling as a result of SCG play. In support, studies have found that adolescents develop inaccurate attitudes regarding monetary gambling through simulated gambling experiences [11]. Specifically, because SCGs aim to maximize player enjoyment in an effort to increase play time and frequency, these games are typically characterized by inflated odds of success alongside augmented payout rates—a system known as dynamic game balancing [82]. Dynamic game balancing in SCGs, therefore, can create the illusion that one is more skilled or perhaps luckier in a gambling game than is actually the case [24]. These flawed beliefs, in turn, prompt players to persist gambling in the face of financial loss when they transition into monetary gambling activities, yielding problematic gambling habits [20, 24, 83]. When these misleading experiences occur early in one’s life, they can be particularly predictive of later pathological gambling [23]. Consequently, the playing of SCGs may be a considerable risk factor for future problem gambling among adolescents.

With that said, it may also be the case that monetary gamblers who exhibit problem gambling tendencies more pervasively engage in social casino gaming [23]. This expansion from monetary gaming into SCGs may be seen as a way to diversify one’s gambling activities, or it may be considered an avenue through which gamblers seek to mitigate their gambling habits by participating in games that simulate gambling activities without carrying the same financial risk [2, 76].

Across all SCGs assessed, the highest proportion of SCG players exhibiting high-severity gambling tendencies were players of simulated slots. This observation is in line with previous studies of monetary gambling, where it has been reported that slot-machine gambling is significantly related to problem gambling among adolescents and adults [84, 85], and tends to be the preferred form of monetary gambling among problem gamblers [86, 87]. This link between slot-machine gambling and problem gambling has been attributed to the structural characteristics of slot-machine games, which are visually stimulating, offer a highly variable reward schedule, and provide instant feedback [88, 89]. Such structural characteristics have been shown to promote frequent and continuous gambling [89]. In the context of SCG play, it is possible that individuals drawn to the SCG of slots also have a preference for engaging in more highly addictive gambling games, such as slot-machine gambling, and may therefore be more likely to exhibit symptoms of gambling problems.

### Factors associated with social casino gaming among adolescents

Overall, the present study identified six factors associated with social casino gaming among adolescents: gender, weekly spending money, smoking status, having friends who gamble, having parents who gamble, and screen time. Although considerable consistency was noted in the factors related to participation in the SCG of slots and SCGs on Facebook, SCG poker players tended to exhibit unique defining characteristics.

In line with previous research, males were more likely than females to report playing the SCG of poker in the past three months, relative to SCG non-players [10]. Gender differences in play were not observed for the SCG of slots or for SCG games on Facebook. The absence of gender differences among Facebook SCG players may be attributable to the fact that simulated gambling games on this social network site are often varied, combining competitive elements that typically appeal to male players with social elements that are typically more attractive to female players [1]. As a result, males and females may be equally drawn to these games. In the case of slots, which tend to be played more pervasively by females in both simulated and monetary forms [10, 32, 33], the rationale for the absence of gender differences is less clear. It is possible that the observed effects stem from the complex associations noted between gender, gambling tendencies, and use of new technologies in past studies. Specifically, while it has been observed that females are more likely to engage in gaming-machine gambling and gambling games of pure chance, such as slots [32], males are more commonly drawn to new digital and online platforms for gaming and gambling [90, 91]. Given that the SCG of slots currently represents a novel technological avenue for gaming, it may be equally appealing to both genders. Additional studies are needed to explore these findings.

Adolescents who reported taking part in SCGs in the past three months, regardless of game type, were more likely to indicate that they receive over $100 per week in allowance or earnings, in comparison to adolescents who did not report using SCGs. These same findings have been observed in studies of social casino gaming and monetary gambling among adults, where higher-income individuals tend to gravitate toward gambling activities [10, 40, 92]. Given that social casino gaming does not require the exchange of currency, it is likely not the case that adolescents with more disposable income are more likely to play SCGs because they would be less financially impacted by gambling losses, as has been suggested for adults [93, 94]. Rather, it may be the case that greater weekly spending among adolescents is an indicator of their household’s higher socioeconomic status. Adolescents residing in more financially stable households may have better access to computer equipment, cellular devices, and technological services, and may therefore have more opportunities to access SCGs [40].

As predicted, it was observed that adolescent SCG players were more likely to report having a friend or a parent who gambles in comparison to adolescents who had not played SCGs the past three months. These results are in line with existing research pertaining to online and land-based gambling [15, 39–41], where it has been noted that close others within an individual’s environment tend to transmit their gambling attitudes and behaviours to the individual, be it through implied approval [37], through modeling [95], or through pressure to conform [96]. The one exception to this general pattern was observed for the SCG of poker, where it was noted that individuals who have played the SCG of poker in the past three months were not more likely to have friends who gamble than individuals who did not report taking part in SCGs. This lack of peer influence among SCG poker players has not been reported in previous investigations, and therefore warrants further study.

In the present study, adolescents reported spending an average of 3.6 hours daily on activities involving screens. This value exceeds the recommendations of the Canadian Sedentary Behaviour Guidelines for Children and Youth, which state that adolescents should limit recreational screen time to no more than 2 hours per day [97]. When sedentary tendencies were assessed in the context of SCG play, it was observed that simulated poker exhibited a unique pattern of associations with screen time in comparison to the other SCG types. Specifically, while it was found that adolescents who spent more time each day watching television, surfing the Internet, or playing computer games were more likely to participate in the SCG of slots and in Facebook SCGs, this same effect was not noted for poker. The finding that screen time may be linked to participation in some social casino gaming is a novel observation that has not been reported in previous assessments of SCG play. Existing research, however, has shown that inactivity and subsequent poor physical health are related to monetary gambling in general [45, 46], and therefore the present findings extend these previous observations. Longitudinal analyses are needed, however, to clarify the causal link between SCG play and sedentary tendencies. It may certainly be the case that individuals with more daily screen time are more likely to be exposed to SCGs, and are therefore more likely to take part in them [11, 17]. At the same time, it is also possible that social casino gaming facilitates sedentary tendencies due to the fact SCGs are typically designed to promote extended play and the frequent return of players to SCG host sites or applications [82]. Further research is also needed to better understand the absence of an association between screen time and the tendency to play the SCG of poker. Some findings from investigations of monetary gambling have noted that recreational poker play tends to be characterized by shorter play times, perhaps due to the taxing nature of the game [98, 99]. As a result, it is possible that participation in the SCG of poker—a form of recreational poker—does not entail considerable daily screen exposure. An empirical assessment of these effects, however, is needed.

In examining substance use in the context of SCG play, only one significant effect was observed. Specifically, it was noted that adolescents classified as current smokers had significantly greater odds of playing the SCG of slots in comparison to former smokers and non-smokers. All remaining types of SCGs were not shown to be linked to substance use. While the non-significant association between binge-drinking and social casino gaming echoes previous results obtained from an assessment of adults [10], the largely absent relationship between tobacco use and social casino gaming stands in contrast to previous findings observed among adults [10]. In studies of monetary gambling, theories of deviance have attributed the typical co-occurrence between gambling and substance use to a general propensity toward risk-taking and the seeking of short-term rewards [59, 94]. These same theories, however, do not appear to extend to SCG play. It may be the case that SCGs are considered insufficiently stimulating for individuals with a propensity toward sensation-seeking, perhaps due to the absence of monetary wins and losses in these games. Instead, these SCGs may attract less impulsive individuals who are not drawn to risky activities, including substance use.

The observation that only the SCG of slots exhibited a significant association with current tobacco use requires further investigation given that it is presently unclear why this particular SCG alone is linked to adolescent smoking status. Given the availability of empirical evidence suggesting that both slot-machine gambling [88, 89] and tobacco use [100] are highly addictive activities, it may be the case that individuals with a tendency to engage in addictive behaviours may seek them out. This explanation is particularly plausible given the considerable structural similarities that exist between the SCG of slots and slot-machine games intended for monetary gambling [76]. Additional research is needed, however, to better understand these findings.

Although existing research pertaining to online gambling suggests that poorer academic performance may be associated with participation in gambling among adolescent samples [15, 43, 44], these effects were not observed in the present investigation across all three SCG types. Rather, social casino gamers did not differ significantly from non-gamers in terms of typical grade achieved in school. Consequently, while achievement in school may be relevant to monetary gambling, it does not appear to be an key factor in defining the profiles of social casino gamers across SCG types.

### Limitations

Although every effort was made to design a study that would yield valid findings, the present investigation is not without its limitations. A central limitation of the study is its cross-sectional design. While this design allowed us to obtain large and representative data that inform the various factors associated with social casino gaming, it prohibits us from stating conclusively that the factors examined are causally predictive of SCG use. The results, however, do demonstrate key links between participation in SCGs and personal and environmental indicators relevant to adolescent populations that have largely been unexamined by previous research endeavours. As a result, the present study offers a foundation for subsequent longitudinal analyses examining predictors of adolescent SCG use.

A further limitation of the present investigation is its reliance on self-report data, which may have been affected by response bias. Specifically, participants may have under-reported specific types of behaviours, especially those deemed socially undesirable [101], although it has been shown that this type of self-management is less problematic for non-pathological gambling behaviours [102]. In an effort to offset potential issues pertinent to self-report assessment in the present study, and to augment honest responding, we collected data through anonymous paper-based questionnaires, and we instructed survey administrators to refrain from moving through the survey location when the measures were being completed by respondents. Although steps were taken to maximize honest responding, the self-report nature of the measures used in the present study did not allow us to collect information about the gambling habits of close others directly from participants’ peers and parents. Future investigations may wish to include other-report measures into their designs in order to obtain primary information from adolescents’ friends and family regarding their own gambling and/or SCG play.

Status of SCG play (players versus non-players) and gambling status (gamblers versus non-gamblers) was limited to the previous three months in the present study. This timeframe was established during the development and validation of the GPSS of the CAGI [66], and this same timeframe was extended to our measures of social casino gaming in the interest of consistency. Due to our restricted focus on the preceding three months, however, it should be noted that the reported results provide a conservative estimate of the effects examined in our study. Additionally, although the present study employed a common threshold of 100 cigarettes to determine smoking status, we acknowledge that this criterion may be arbitrary, and perhaps too coarse to effectively capture the complex nature of early experimentation with tobacco among adolescents [70]. Furthermore, we recognize that Facebook better reflects a medium for accessing SCGs rather than a type of SCG, and therefore our assessment of SCGs on Facebook may have confounded point of access with game class. Future studies of SCGs are encouraged to assess SCG types independently from SCG mediums.

Due to the fact that the YGS was a supplementary measure with length restrictions, it was not feasible to assess an exhaustive list of variables potentially relevant to SCG play. Variables that were not examined in the present study, but that may ultimately bear relevance to assessments of SCG player characteristics include: platform of access to SCGs (e.g., social networks, apps, video games, demo games [28], location of access to SCGs (e.g., home versus outside of home) [28], an exhaustive list of all possible SCGs available to players (e.g., sports betting, bingo, blackjack) [1], and frequency of SCG play [30]. Subsequent investigations of SCGs are encouraged to examine these variables in an effort to yield a more comprehensive understanding of social casino gaming, particularly among adolescents.

## Conclusion

With the increasing convergence between gambling and gaming activities online, as seen in part through the proliferation of SCGs across various web-based platforms, individuals are being increasingly prompted to engage in simulated gambling behaviours [38]. This early exposure to gambling may be particularly detrimental to adolescents, who are especially avid users of SCGs, given that it may be a risk factor in subsequent monetary and problem gambling, as suggested in the present study and as shown in previous research [14, 24]. However, it should also be noted that the transition from SCG exposure to subsequent gambling may be complex, and influenced by numerous biological, social, behavioural, cognitive, emotional, and motivational factors [23, 24]. The present study identified some factors that appear to be associated with SCG play among adolescents, including gender, weekly spending money, smoking status, having friends who gamble, having parents who gamble, and screen time. Of these factors, the social influences of peers and parents have been named as both risk and protective factors in the transition from social casino gaming to monetary gambling in existing path models [23]. Specifically, while the monitoring of adolescent social casino gaming by parents has been identified as a protective factor that inhibits the transition from SCG play to monetary gambling, peer pressure to gamble and the modeling of irresponsible gambling behaviour by parents have been classified as catalysts in this same transition. Based on the present results, existing path models may wish to expand their scope to include the socioeconomic status and health behaviours of players to provide a more comprehensive overview of the manner in which SCG play and monetary gambling may be linked. At the same time, future studies of SCG play and monetary gambling may wish to examine additional factors related to these activities, as identified in existing path models.

In aiming to develop and tailor intervention and awareness strategies aimed at mitigating the potentially negative influence of SCGs on adolescents, involved parties should be cognizant of the personal and environmental factors that have been implied in social casino gaming among this cohort, including gender, availability of disposable income, presence of peers or parents with gambling tendencies, sedentary propensities and, to a lesser extent, tobacco use. Further, it should be noted that generalized educational or intervention efforts aimed at addressing all social casino gaming among adolescents may be less effective than programs that recognize the unique profiles of individuals who engage in different types of SCGs. Longitudinal research that explores the causal links between SCG habits and the factors implicated in SCG play in the current study are needed.

## Abbreviations

CSTADS: Canadian Student Tobacco, Alcohol and Drugs Survey
SCG: Social casino game
YGS: Youth Gambling Survey
YSS: Youth Smoking Survey
